# Analysis of Microbial Community Diversity on the Epidermis of Wine Grapes in Manasi’s Vineyard, Xinjiang

**DOI:** 10.3390/foods11203174

**Published:** 2022-10-12

**Authors:** Xiaoyu Xu, Yuanyuan Miao, Huan Wang, Juan Du, Chenqiang Wang, Xuewei Shi, Bin Wang

**Affiliations:** 1 Food College, Shihezi University, Shihezi 832000, China; 2 Xinjiang Sailimu Modern Agriculture Co., Ltd., Shuanghe 833408, China; 3 Guannong Testing Technology Co., Ltd., Tiemenguan 841007, China

**Keywords:** wine grapes, grape surface microorganisms, Illumina high-throughput sequencing, microbial diversity

## Abstract

Epiphytic microbial communities significantly impact the health and quality of grape berries. This study utilized high-performance liquid chromatography and high-throughput sequencing to explore the epiphytic microbial diversity and physicochemical indicators in nine different wine grape varieties. In total, 1,056,651 high-quality bacterial 16S rDNA sequences and 1,101,314 fungal ITS reads were used for taxonomic categorization. Among the bacteria, Proteobacteria and Firmicutes were the dominant phyla, and *Massilia*, *Pantoea*, *Pseudomonas*, *Halomonas*, *Corynebacterium*, *Bacillus*, *Anaerococcus,* and *Acinetobacter* were the dominant genera. Among the fungi, Ascomycota and Basidiomycota were the dominant phyla, and *Alternaria*, *Filobasidium*, *Erysiphe*, *Naganishia*, and *Aureobasidium* were the dominant genera. Notably, Matheran (MSL) and Riesling (RS) exhibited the highest microbial diversity among the nine grape varieties. Moreover, pronounced differences in epiphytic microorganisms in red and white grapes suggested that the grape variety significantly influences the structure of surface microbial communities. Understanding the composition of epiphytic microorganisms on the grape skin can provide a direct guide to winemaking.

## 1. Introduction

Wine, an alcoholic beverage, is prepared via the microbial fermentation of grapes or grape juice using microorganisms, such as *Saccharomyces cerevisiae* [1]. Based on the color, wine is classified as red, white, or rose wine [2,3]. Cabernet Sauvignon, Merlot, Pinot Noir, and Syrah are the main grape varieties used for red wines, while Riesling, Chenin Blanc, and Chardonnay are mostly used for white wines. Different grape varieties provide distinctive characteristics, such as flavor (e.g., fruity flavor), tannins, acidity, and alcohol contents, that affect the wine quality [4,5].

Microorganisms are essential to the winemaking process. They not only drive fermentation but also significantly influence the flavor, aroma, quality, and value of the wine [6,7]. In the winemaking process, grape berries are the main source of microbial communities and, therefore, the microbial diversity of wine grapes has attracted serious attention. A variety of complex microorganisms (such as yeasts, molds, and bacteria) on the skin of ripe grape berries contribute to the formation of wine flavor [8]. Geography, soil status, climate, and cultivation conditions significantly influence the structure of microbial communities [9]. For instance, fruit microflora adapted to local geographical, climatic, and other conditions of a grape wine-producing region provides a specific flavor and quality to the wine of that region [3]. Xinjiang (China) is one such premium wine grape region with unique climatic and superior geographical conditions [10]. Specifically, Manasi County in Xinjiang has gravel sandy loam soil, long sunshine hours, a large temperature difference between day and night, and low precipitation, all of which help make it a premium wine-producing area.

High-throughput sequencing (HTS) and multivariate data analysis have become popular methods for studying microorganisms in a variety of contexts, including food and gut microbes [11]. Traditional microbial culture methods provide limited insights into microbial diversity, whereas HTS allows for the study of microbes that cannot be cultured or are yet to be recognized. HTS helps to understand microbial diversity on a much larger scale by collecting large, high-precision, and low-cost data [12]. HTS has also been used to study microbial diversity in grapes and wines from different regions [13] and vintages [14] and derive comparisons between different wine styles. Moreover, such studies concluded that wine microbes are the main determinants of wine quality and sensory style [15]. An HTS study revealed the presence of *Gluconobacter* in Chardonnay wine from two different regions in California, USA; *Gluconobacter* produces acetic acid during the winemaking process, which adversely affects the organoleptic quality of the wines [16]. A study showed that a change in microbial communities on the surface of Grenache and Carignan grapes from vineyard to vineyard affected the grape quality, even in close geographical locations, highlighting the role of the microbial population in wine production [3]. Likewise, another study showed that the structural characteristics of the grape/wine microbial community can be highly specific to a region [17]. Gao et al. [18] found that *Aureobasidum*, *Cryptococcus*, *Hanseniaspora*, *Alternaria*, *Rhodotorula*, *Botrytis*, *Mucor*, *Chaetopyrena*, *Cladosporium*, and *Fusarium* were the dominant fungal genera on the grape surface of four wine grapes (Cabernet Sauvignon, Merlot, Italic Riesling, and Cabernet Franc) in the wine-producing areas of northern Xinjiang, China (e.g., Shanshan, Yanqi, and Heshuo regions). All these studies indicated that a variety of factors influence the composition and structure of grape surface microbiota.

Microbial diversity and its control during wine fermentation have gained growing attention [19]. However, the variables that influence the diversity of wine grape surface microbiota remain poorly understood. This study used HTS to characterize the microbial communities on the grapevine carposphere of nine (five red and four white) wine grape varieties. Our results revealed the microbial diversity in Xinjiang vineyards and set a theoretical basis for the screening and establishment of high-quality winemaking microorganisms.

## 2. Materials and Methods

### 2.1. Sampling Site

This study was conducted in a large vineyard in Manas County, Changji Hui Autonomous Prefecture, Xinjiang Uygur Autonomous Region (44.18′ N, 86.13′ E). The region has a temperate continental arid climate, with long and harsh winters, short and hot summers, and an average annual temperature of 7–8 °C. The vineyards did not involve endangered or protected species, are not privately owned or protected, and did not require any special permits. The vines were 10 to 37 years old.

### 2.2. Grape Sampling

The fruit was collected from nine varieties of wine grapes, including five red (CS, Cabernet Sauvignon; SR, Syrah; PN, Pinot Noir; ML, Merlot; MS, Marselan) and four white (RS, Riesling; CB, Chenin blanc; IR, Italian Riesling; EL, Ecolly) wine grape varieties. Disease-free and fresh wine grape fruit samples were collected using scissors that were disinfected with 75% alcohol. The collected samples were put in sterile self-sealing bags, transported back to the laboratory in low-temperature boxes, and then a portion of the samples was frozen at −20 °C as a backup [20]. For each wine grape variety, the “five-point sampling approach” was used to gather samples from five plants, which were then combined for further tests.

### 2.3. Determination of Physicochemical Properties

Physiochemical indicators, including pH, total sugars (TS), total acidity (TA), and soluble solids content (SSC), of the grape samples were measured. The total sugar content was determined using the dinitro salicylic acid method, and the pH was measured using a calibrated pH meter [21]. The total acid content was calculated following the national standard GB/T 15038-2006 “General analytical procedure for wine and fruit wines”.

Organic acids and soluble sugars were determined using high-performance liquid chromatography (HPLC). Soluble sugars were extracted according to Cao et al. [22] with some modifications. The chromatographic separation of sugars was performed with an XBridge amide column (5 μm, 4.6 mm × 250 mm), and acetonitrile and water were used as the mobile phases at a flow rate of 1 mL/min. The injection volume was 20 μL, the column temperature was 30 °C, and the elution peak was detected using an RID-10A differential detector.

Organic acids were estimated following the method of Ryan et al. [23] with some modifications. Grape samples were centrifuged and filtered into injection vials using 0.45 μm filters. The other experimental conditions were as follows: chromatographic column, Dikma C18 column (5 m, 4.6 mm, 250 mm; Diamonsil Plus Technology, China); column temperature, 40 °C; mobile phase, 0.1% phosphoric acid and methanol; flow rate, 0.7 mL/min; UV detection, 210 nm. A calibration curve was plotted using standards to determine the peak area versus concentration.

### 2.4. Microbial Diversity Analysis

The grape samples were stemmed to avoid contamination by endophytic bacteria inside the grapes. Each sample of wine grape variety was weighed to approximately 30 g, added to 100 mL PBS buffer (0.1 mol/L, pH 7.0), and vortex shaken for 30 min at 180 r/min. After sonication for 15 min, the membrane was filtered with a 0.22 μm microporous membrane (Millipore, Burlington, MA, USA), cut, and placed in a sterilized centrifuge tube for total DNA extraction.

Total genomic DNA samples were extracted using the OMEGA Soil DNA Kit (M5635-02) (Omega Bio-Tek, Norcross, GA, USA) following the manufacturer’s instructions. The extracted DNA samples were assessed for quantity and quality using spectrophotometry and agarose gel electrophoresis. The fungal ITS1 region was PCR amplified using the forward primer ITS5F (5′-GGAAGTAAAAGTCGTAACAAGG-3′) and the reverse primer ITS1R (5′-GCTGCGTTCTTCATCGATGC-3′). The V3–V4 region of the bacterial 16S rRNA gene was PCR amplified using the forward primer 338F (5′-ACTCCTACGGGAGGCAGCA-3′) and the reverse primer 806R (5′-GGACTACHVGGGTWTCTAAT-3′). The PCR conditions were as follows: denaturation at 98 °C for 5 min, followed by 25 cycles of annealing at 53 °C for 30 s and extension at 72 °C for 45 s, and a final extension at 72 °C for 5 min [24]. The obtained PCR-amplified products were sequenced on an Illumina MiSeq platform at Shanghai Personal Biotechnology Co. QIIME2 2019.4 with some minor modifications to the protocol specified in the official tutorial (https://docs.qiime2.org/ accessed on 1 August), and the data were subjected to microbial bioinformatics.

### 2.5. Data Analysis

In this investigation, three parallel tests were run for each grape sample. The experimental data were analyzed for the significance of variance using SPSS data processing software (version 20; IBM, Chicago, IL, USA) at a significance level of *p* < 0.05. Origin 2021 was used to generate bar graphs. Principal component analysis (PCA) plots were drawn using SIMCA 14.1 software based on the Bray–Curtis distance algorithm to analyze the distribution patterns of microbial communities in different grape samples. Chord diagrams and heat maps were prepared using R (version 3.3.1) [25].

## 3. Results and Discussion

### 3.1. Changes in Physicochemical Characteristics

#### 3.1.1. General Physiochemical Indicators

**Figure 1:**
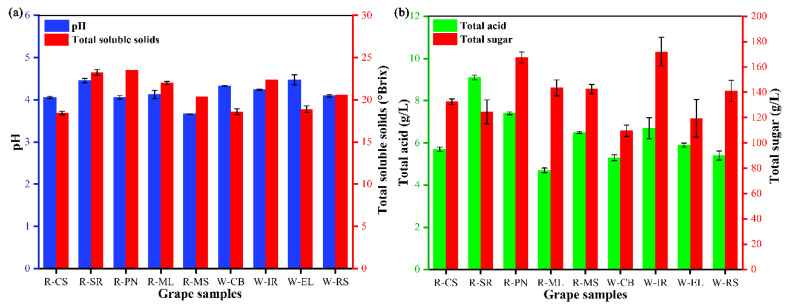
Overall differences in pH, soluble solids content (**a**), total sugars, and total acidity (**b**) of different wine grapes.

Physicochemical characteristics define the wine grape quality. The basic physicochemical indicators, such as pH, TA, TS, and SSC, of the ripening fruit of the wine grape varieties are shown in Figure 1. Climate and other factors influence fruit ripening, altering the physicochemical indices of different wine grape varieties from the same origin [26]. For the nine grape varieties, the pH ranged between 3.96 and 4.22, and the SSC ranged between 18.45 and 23.5° Brix. Among all the grape varieties, CS had the lowest SSC content. Among the white grape varieties, IR had the highest SSC. The TA content ranged between 4.7 and 9.1 g/L, showing significant differences between the wine grape varieties (*p* < 0.05). ML grapes had the lowest TA content (7.14 g/L), while others (except SR) had a TA content <7.40 g/L. The highest TS content was found in the white wine grape variety IR, followed by the red wine grape variety PN. CB had the lowest TS content of 109.5 g/L.

#### 3.1.2. The Compositions and Contents of Sugar and Organic Acids

**Figure 2:**
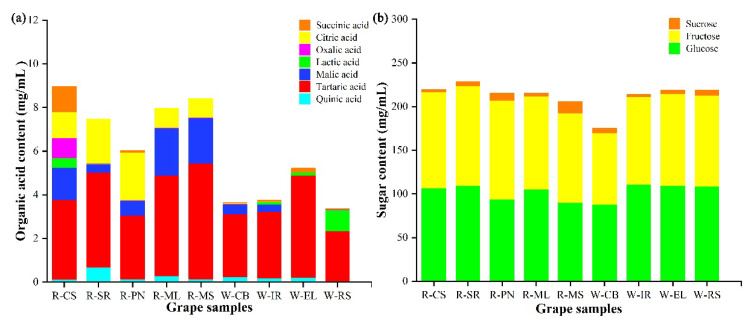
Differences in the organic acids (**a**) and soluble sugars (**b**) in different wine grape samples.

Organic acids are natural components in grapes and affect the color, flavor, aroma, and other characteristics of grape wine [27,28]. Grape organic acids are the key products of fruit metabolism, and their distribution and concentration characteristics are closely associated with the processing of wine grapes [29]. In this study, seven organic acids (succinic, citric, oxalic, lactic, malic, tartaric, and quinic acids) were detected in wine grapes (Figure 2a). Consistent with Lima et al. [30], tartaric and malic acids were found to be the main organic acids in grapes. Their sum content accounted for >80% of the total organic acids in all grape varieties. The highest levels of tartaric (5.3 g/L) and malic (2.71 g/L) acids were present in SR and ML grapes. Except for CS, white grape varieties typically had higher levels of lactic acid than red grapes, while the opposite was true for oxalic acid. Consistent with Haggerty [31], succinic and quinic acids were in lower amounts. Several factors such as grape variety, climate, and stage of ripening can vary the content of organic acids in wine grapes.

SSC in grapes directly affects yeast reproduction and metabolism ultimately affecting the quality of wine alcohol [32]. The components and contents of soluble sugars in nine wine grapes were analyzed using HPLC (Figure 2b). Fructose, glucose, and sucrose were in the ranges of 60.00–114.20, 63.00–109.56, and 3.06–12.98 g/L, respectively. Glucose and fructose were the main sugars in all grapes. Among the red grape varieties, SR had the highest glucose and fructose content, while CB had the lowest glucose and fructose content among the white grape varieties. We found no significant differences in the fructose and glucose contents among the other grape varieties, except for CB. Consistent with Liu et al. [33], who reported sucrose levels in the range of 0.12–2.83 mg/mL, we also found sucrose in small amounts in our grape samples, while no maltose was detected.

### 3.2. Microbial Succession and Interactions

#### 3.2.1. Sequencing Quality Assessment

**Figure 3:**
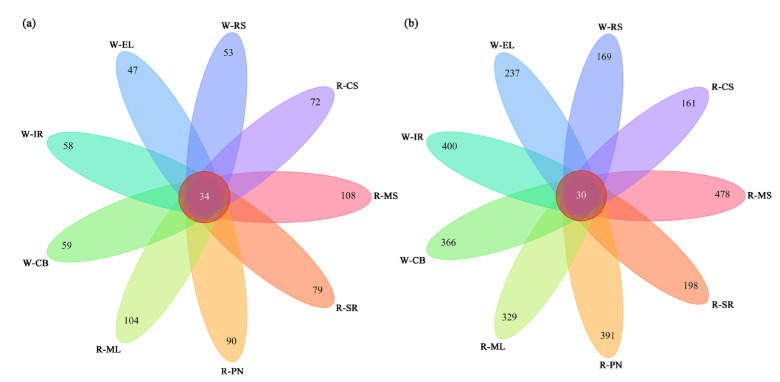
Venn diagrams of the fungal (**a**) and bacterial (**b**) OTUs among different wine grape samples.

We employed HTS to study the diversity of epidermal microbial communities in wine grapes. Poor-quality sequences and chimeras were eliminated to obtain valid sequences from each sample. In total, 1,056,651 high-quality bacterial sequences and 1,101,314 fungal sequences were obtained from all the samples. The fungal sequences outnumbered the bacterial sequences. For all samples, the coverage of high-quality sequences was >99%, and the flat coefficient curves indicated an adequate diversity of sequences (Figures S1 and S2). Additionally, the sequencing results indicated that our sequencing data had an appropriate volume. After data normalization, we performed α-diversity analysis for species richness and diversity, such as the Chao1 and Shannon indices (Table S1). Concerning bacterial communities, MS showed the highest richness, as indicated by the Chao1, Shannon, and Simpson indices (Figure S3). Regarding the fungal communities, ML grapes were richer in both abundance and diversity than other grapes (Figure S4). These results highlighted the variation in richness and abundance of epiphytic microbial communities in different grape varieties. Totals of 72, 79, 90, 104, 108, 59, 58, 47, and 53 OTUs for fungal communities and 161, 198, 391, 329, 478, 366, 400, 237, and 169 OTUs for bacterial communities were recorded in the CS, SR, PN, ML, MS, CB, IR, EL, and RS grapes, respectively (Figure 3). Interestingly, apart from a shared microbial community, several wine grape varieties also had unique microbial communities.

#### 3.2.2. Sequencing Quality Assessment

We further analyzed the diversity and community succession of grapevine epidermis microorganisms. Based on the Illumina sequencing of 27 samples from nine grape varieties, we obtained the number of fungal and bacterial taxonomic units at each taxonomic level (Figure S5). At the genus level, the highest and lowest numbers of bacterial taxonomic units were detected in MSL and RS samples, respectively. The highest number of fungal taxonomic units was detected in ML grapes.

**Figure 4:**
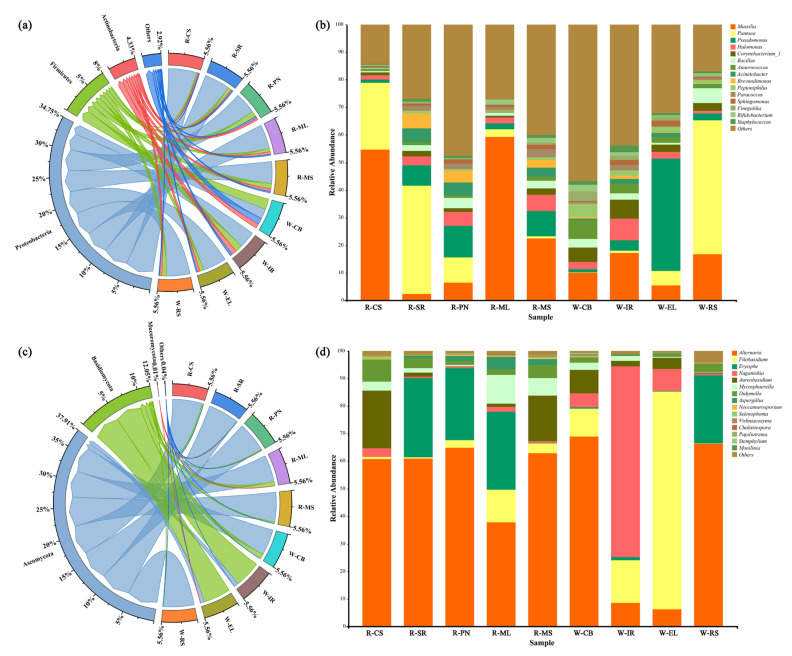
Differences in fungal (**a**) and bacterial (**c**) communities at the phylum level. The relative abundances of the top 15 fungi (**b**) and bacteria (**d**) at the genus level.

Moreover, the main phyla and genera were present in all grape varieties, though in varying degrees. At the phylum level, Proteobacteria (including nitrogen-fixing bacteria and rhizobacteria) predominated in the bacterial population of all grape samples (Figure 4b) [34]. Proteobacteria were followed by Firmicutes, Actinobacteria, etc. The Firmicutes include various fermentation bacteria [35]. Studies showed that vineyard soils and leaves are equally dominated by Proteobacteria, Firmicutes, and Actinobacteria and these microorganisms are prevalent in the environment [36,37]. *Massilia*, *Pantoea*, *Pseudomonas*, *Halomonas*, *Corynebacterium*, *Bacillus*, *Anaerococcus*, *Acinetobacter*, *Brevundimonas*, *Peptoniphilus*, *Paracoccus*, *Sphingomonas*, *Finegoldia*, *Bifidobacterium*, and *Staphylococcus* were the 15 main bacterial genera in the nine wine grape varieties (Figure 4d). Notably, *Massilia* was the most abundant bacterial genus in CS and ML grapes, while the *Pantoea* genus was abundant in SR and RS grapes. Some studies found *Bacillus* and *Pantoea* genera on ripe berries, which are also present in the soil [18]. Therefore, these results indicate an ecological link with the epiphytic communities in the subterranean part of the plant [38]. In general, except for a few pathogens, the microbiota is usually beneficial to the host. *Bacillus* is most frequently used as a biocide in viticulture to control fungal infections [39]. *Acinetobacter*, which is a group of heterotrophic nitrifying bacteria, has a denitrification capacity and contributes to nitrogen fixation in plants [40]. *Pseudomonas* bacteria are typically present in vineyard soils and during wine fermentation but their origin is poorly known [41]. Our results suggested that they are from the grape surface.

In comparison to bacterial communities, the diversity among grape fungi was significantly lower. Consistent with Singh et al. [42], Ascomycota, followed by Basidiomycota, dominated the fungal communities at the phylum level in all nine grape varieties (Figure 4a). At the genus level, the dominant fungi on grape skins were *Alternaria*, *Filobasidium*, *Erysiphe*, *Naganishia*, and *Aureobasidium*. *Alternaria* dominated all grape skins except IR and EL (Figure 4c). *Alternaria* is considered one of the primary fungal populations during grape harvest [34], which acts as a biotrophic pathogen (latent in the fruit’s outer layer) and infects the fruit during development and flowering [43]. Botrytis, which is a necrotrophic fungal infection, was not found in this study, possibly due to the study location [44]. In general, healthy plants attempt to maintain pathogenic bacterial populations at a minimum level. Accordingly, *Saccharomyces cerevisiae* was not detected in our grapes, indicating the least infection in healthy and undamaged grapes [45].

#### 3.2.3. Cluster Analysis

**Figure 5:**
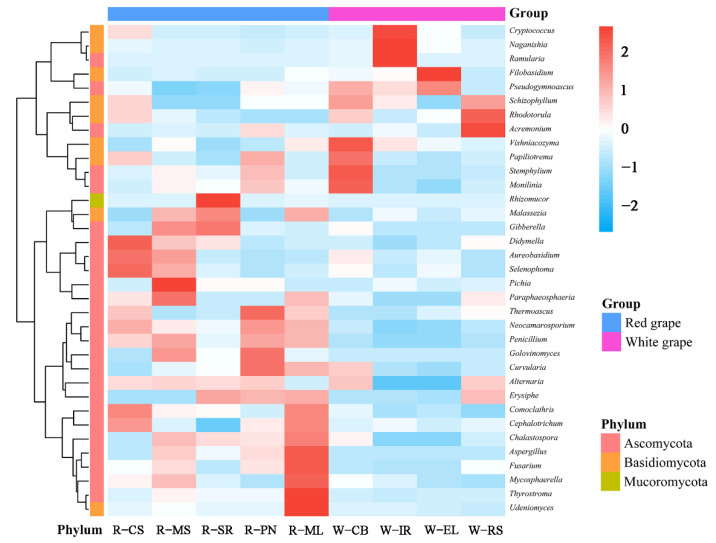
Heatmap of the top 35 abundant fungal genera in different grape samples. Samples are clustered according to the similarity between their constituents and arranged in horizontal order. Red and blue represent the more and less abundant genera in the corresponding group, respectively.

The grape samples were clustered according to the species abundance information and heat maps were produced. In total, 35 fungal genera were in high abundance. The clustering heat map showing the relative abundance of fungi is shown in Figure 5. The different grape varieties showed significant variation in fungal composition. *Didymella*, *Aureobasidiu*, and *Selenophoma* were the dominant strains in CS grapes. Among all grapes, ML showed the highest microbial diversity with the highest abundances found for *Thyrostroma* and *Udeniomyces*. *Pichia* showed the highest abundance in MS. *Pichia* is frequently present in fermented foods (such as wine) and promotes their flavor by producing enzymes during metabolic activity [46,47]. *Cryptococcus* and *Rhodotorula* showed a relatively high abundance in IR and RS, respectively. *Vishniacozyma*, which is a dominant species in the grape skins of Xinjiang’s organic vineyards and the ice wine made in Yili, Xinjiang, China, was in relatively high abundance in CB grapes [48]. *Vishniacozyma* was isolated from a variety of substrates, including wood [49], soil (including Antarctic, Alaskan, and Arctic soils) [50], and cold settings [51]. Its potential impact on wine quality, particularly on wine flavor, is currently unknown and requires further research.

**Figure 6:**
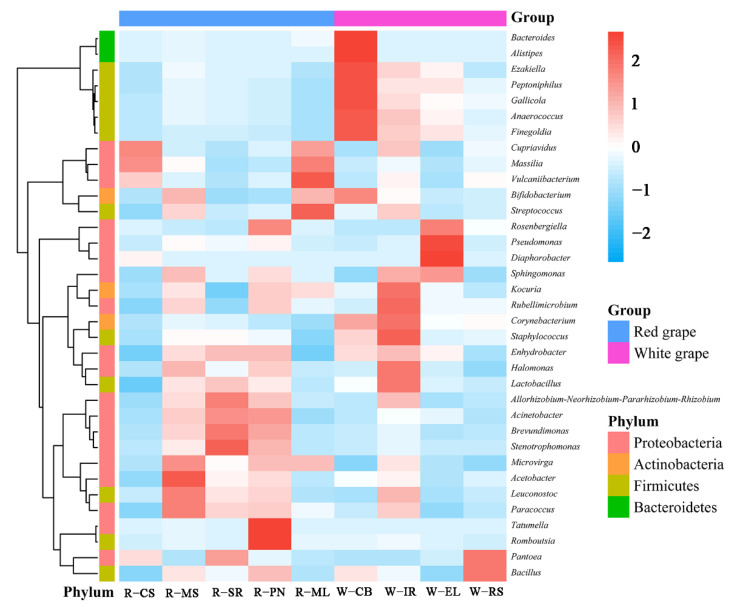
Heatmap of the top 35 abundant bacterial genera in different grape samples. Samples are clustered according to the similarity among their constituents and arranged in horizontal order. Red and blue represent the more and less abundant genera in the corresponding group, respectively.

The heatmap clustering for bacteria is shown in Figure 6. Bacterial communities greatly vary depending on the wine grape variety. *Pantoea* and *Bacillus* had a relatively high abundance in RS grapes. *Pantoea* is a widely existing bacteria on the surface of plants, grains, and fruits [52,53]. *Lactobacillus*, which is a key bacteria in wine manufacture, was in the highest relative abundance in IR grapes. It produces lactic acid and numerous antibacterial compounds (such as bacteriocins) during the wine-brewing process and suppresses the growth of pathogens and spoilage microorganisms [54]. *Massilia* bacteria were predominantly found in Chardonnay by Leveau and Tech [55]. We too found a higher abundance of *Massilia* in ML and CS grapes. *Sphingomonas* can survive wine fermentation but its effect on wine sensory properties is unknown [48]. Studies suggested that at the genus level, bacterial communities may be directly affected by the external environment [56], temperature, and other factors, resulting in different bacterial community compositions in different grape varieties.

**Figure 7:**
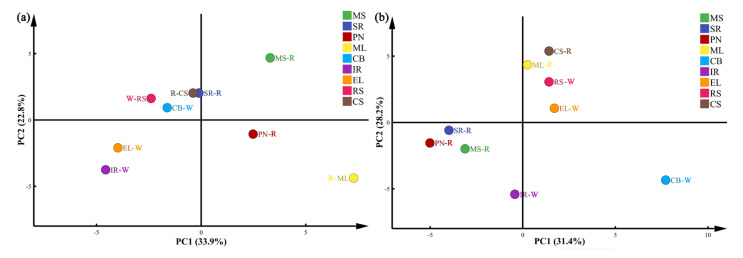
Principal component analysis (PCA) scatter plot of the fungal (**a**) and bacterial communities (**b**) in the samples.

We next performed principal component analysis (PCA) to compare the differences and similarities of microbial diversity between different grape samples. Our PCA-based analysis of the fungal communities revealed that the first (PC1) and second (PC2) principal components respectively accounted for 43.2 and 25.1% of the variations between samples (Figure 7a). For bacteria, the contribution rates of PC1 and PC2 were 31.4 and 28.2%, respectively (Figure 7b). Our results suggested that despite being grown similarly and in the same region, the wine grape varieties showed significant differences in species diversity. Notably, we found closer proximity between the samples of red grape varieties compared with white grape varieties. This indicated the similar microbial compositions between grape varieties of the same color. Additionally, these results also suggested that genotyping (cultivar) affects the variety and make-up of microbial species in grape skins [42].

### 3.3. Co-Occurrence Analyses for Relationships between Different Microbes

**Figure 8:**
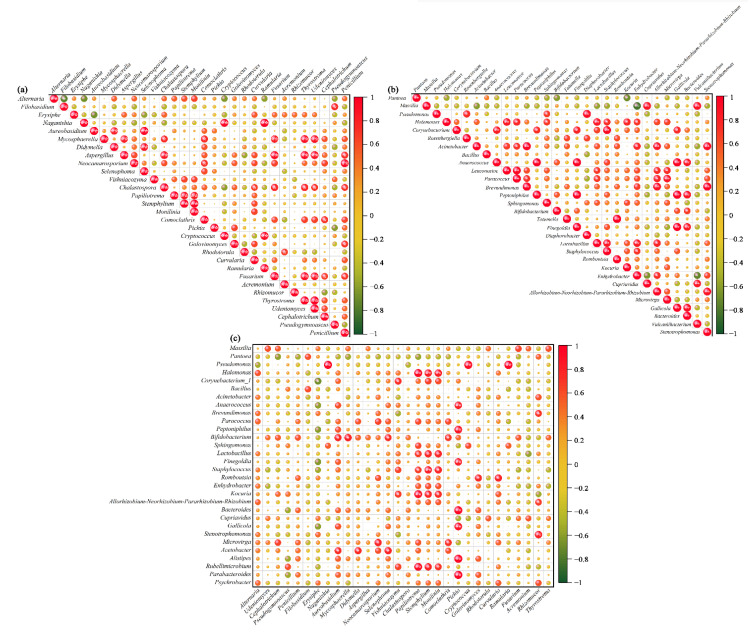
Co-occurrence and co-exclusion relationships between different bacteria (**a**) and fungi (**b**) and between bacteria and fungi (**c**). The Pearson rank correlation matrix showing the abundances of the top 30 fungi and bacterial genera is depicted. Strong and weak correlations are indicated by the large and small circles, respectively. The color of the scale bar denotes the nature of the correlation; 1 indicates a perfect positive correlation (**red**) and −1 indicates a perfect negative correlation (**green**). Significant correlations (|r| > 0.7, *p* < 0.01) and (|r| > 0.9, *p* < 0.01) are indicated by * and **, respectively.

Microorganism interactions play a key role in maintaining the structure of the microbial community [11]. To examine the possible association between the dominant microorganisms, we performed a Pearson rank correction coefficient analysis (Figure 8). Among the fungi, *Aspergillus* and *Mycosphaerella* showed a co-occurrence with *Fusarium*, *Thyrostroma*, and *Udeniomyces*. There was a negative correlation between *Alternaria* and *Filobasidium* (Figure 8a). Concerning bacteria, *Gallicola* and *Bacteroides* were positively correlated with *Anaerococcus*, *Peptoniphilus*, and *Finegoldia*. *Massilia* showed exclusion with *Enhydrobacter* (Figure 8b). In addition, correlation analysis between bacteria and fungi indicated that *Pappiliotrema*, *Stemphylium*, and *Monilinia* were positively correlated with *Halomonas*, *Lactobacillus*, *Staphylococcus*, *Kocuria,* and *Rubellimicrobium*. *Pichia* also showed a positive association with *Peptoniphilus*, *Finegoldia*, *Bacteroides*, *Gallicola, Alistipes*, and *Parabacteroides* (Figure 8c). Although the relative abundance of some microbial groups was not high, they could still play a key role in maintaining the stability of the grape skin microbial community. A close association between certain microorganisms can have a larger impact on the structural composition and functional changes in their communities [57]. Overall, our results offer fresh perspectives on microbial management for viticulture and winemaking.

## 4. Conclusions

Specific microorganisms from the Xinjiang region play an important role in wine production as indigenous fermenters. HTS was used to examine the microbial diversity among the nine different grape varieties from Manasi county, Xinjiang, China, and the dominant genera and phyla were revealed. The grape varieties (cultivars) influenced the microorganisms on their berries, which consequently affected the fruit quality. Wine grape skin microbes significantly impact the wine-making process and add distinctive aromas to wines with regional peculiarities. In the future, we plan to study the relationship between functional grape skin microorganisms and the wine flavor.

## Data Availability

Data are contained within the article.

## Supplementary Materials

The following supporting information can be downloaded from https://www.mdpi.com/article/10.3390/foods11203174/s1. Table S1: Richness and diversity indices of fungal and bacterial communities among different grape samples; Figure S1: Assessment of sparse curves for fungal sequencing saturation; Figure S2: Assessment of sparse curves for bacterial sequencing saturation; Figure S3: Alpha diversity indices for fungal communities; Figure S4: Alpha diversity indices for bacterial communities; Figure S5: Statistics of the microbiota at each classification level for fungi (a) and bacteria (b).
