# Supplementary material for: Analysis of Microbial Community Diversity on the Epidermis of Wine Grapes in Manasi’s Vineyard, Xinjiang
Source: Foods. 2022 Oct 12;11(20):3174. doi: 10.3390/foods11203174 (PMC9602134; doi:10.3390/foods11203174)
Supplement: Supplementary file 1 [file foods-11-03174-s001.zip › foods-1948292-supplementary.pdf]

Catalog

Supplementary Table...1

Table S1. Richness and diversity indices of fungal and bacterial communities among different grape samples...1

Supplementary figures...1

Figure S1. Assessment of sparse curves for fungal sequencing saturation. 1

Figure S2. Assessment of sparse curves for bacterial sequencing saturation..................................2

Figure S3. Alpha diversity indices for fungal communities..3

Figure S4. Alpha diversity indices for bacterial communities. 4

Figure S5. Statistics of the microbiota at each classification level for fungi (a) and bacteria (b)...5

Supplementary Table

Table S1. Richness and diversity indices of fungal and bacterial communities among different grape samples.

Samples	Fungi				Bacteria			
	Chao1	Observed species	Shannon	Simpson	Chao1	Observed species	Shannon	Simpson
R-CS	129.30±8.32^{c}	128.00±15.23^{c}	2.01±0.14^{b}	0.54±0.13^{b}	263.98±18.42^{f}	143.76±13.25^{c}	4.84±0.07^{d}	0.91±0.11^{c}
R-SR	135.24±10.12^{b}	133.60±14.20^{b}	1.73±0.02^{c}	0.54±0.11^{b}	393.88±14.23^{e}	216.33±10.20^{b}	5.90±0.03^{c}	0.95±0.23^{b}
R-PN	129.60±16.22^{c}	128.12±8.32^{c}	1.65±0.11^{c}	0.52±0.06^{b}	740.47±12.74^{b}	343.36±12.20^{a}	6.69±0.02^{b}	0.95±0.09^{b}
R-ML	168.66±17.62^{a}	168.03±16.23^{a}	2.75±0.12$_{a}$	0.74±0.15^{a}	420.47±15.44^{d}	225.12±12.63^{b}	5.88±0.01^{c}	0.94±0.08b^{c}
R-MS	164.76±15.63^{a}	162.41±15.32^{a}	2.17±0.11^{b}	0.54±0.01^{b}	950.94±15.23^{a}	398.41±10.36^{a}	7.26±1.23^{a}	0.97±0.08^{a}
W-CB	128.60±14.23^{c}	126.66±14.20^{c}	1.89±0.02^{c}	0.45±0.03^{c}	448.42±9.56^{d}	280.86±11.45^{b}	7.09±0.42^{a}	0.98±0.07^{a}
W-IR	102.69±18.63^{d}	101.61±12.32^{d}	2.67±0.04^{a}	0.71±0.13^{a}	543.37±6.32^{c}	321.13±6.50^{a}	7.20±0.12^{a}	0.98±0.04^{a}
W-EL	81.10±7.64^{f}	80.73±10.32^{e}	1.37±0.21^{d}	0.33±0.08^{d}	316.99±7.23^{e}	194.26±13.98^{c}	5.14±0.36^{c}	0.85±0.09^{d}
W-RS	91.23±9.82^{e}	90.46±14.23^{f}	1.43±0.14^{d}	0.48±0.09^{c}	275.77±6.23^{f}	169.03±2.35^{c}	5.14±0.41^{c}	0.91±0.01^{c}

Different superscripts indicate significant differences between samples ($p < 0.05$, Duncan's test).

Supplementary figures

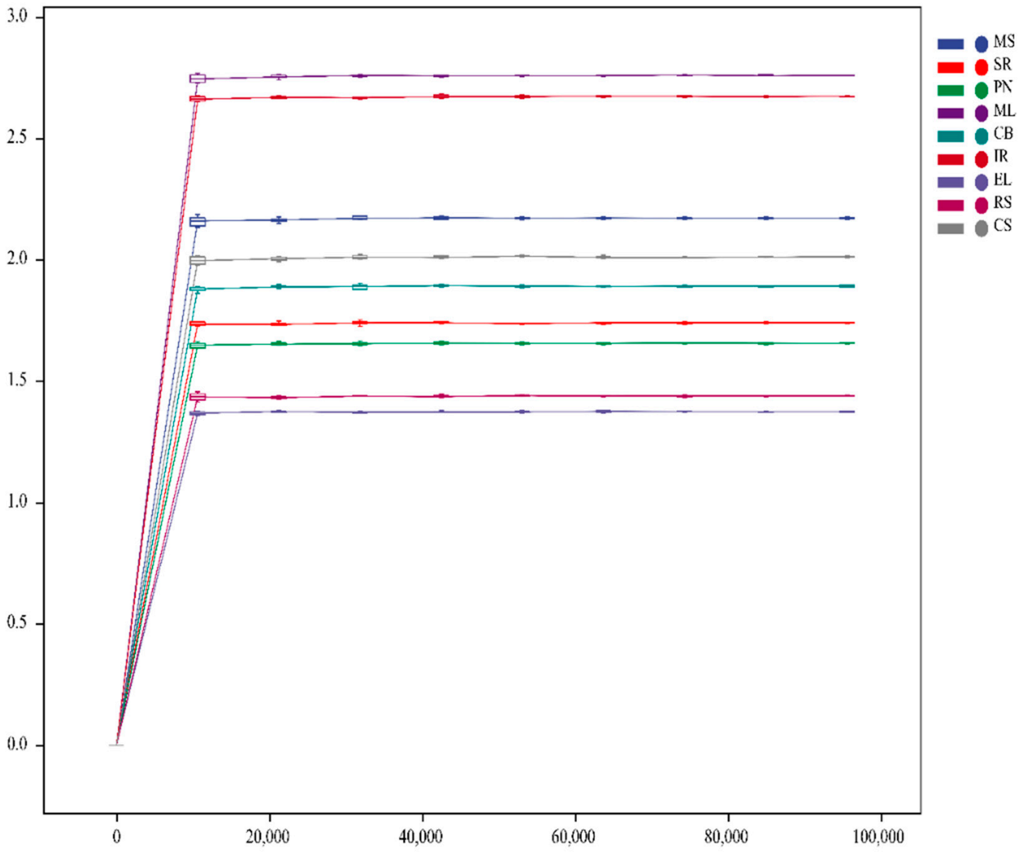

Figure S1. Assessment of sparse curves for fungal sequencing saturation.

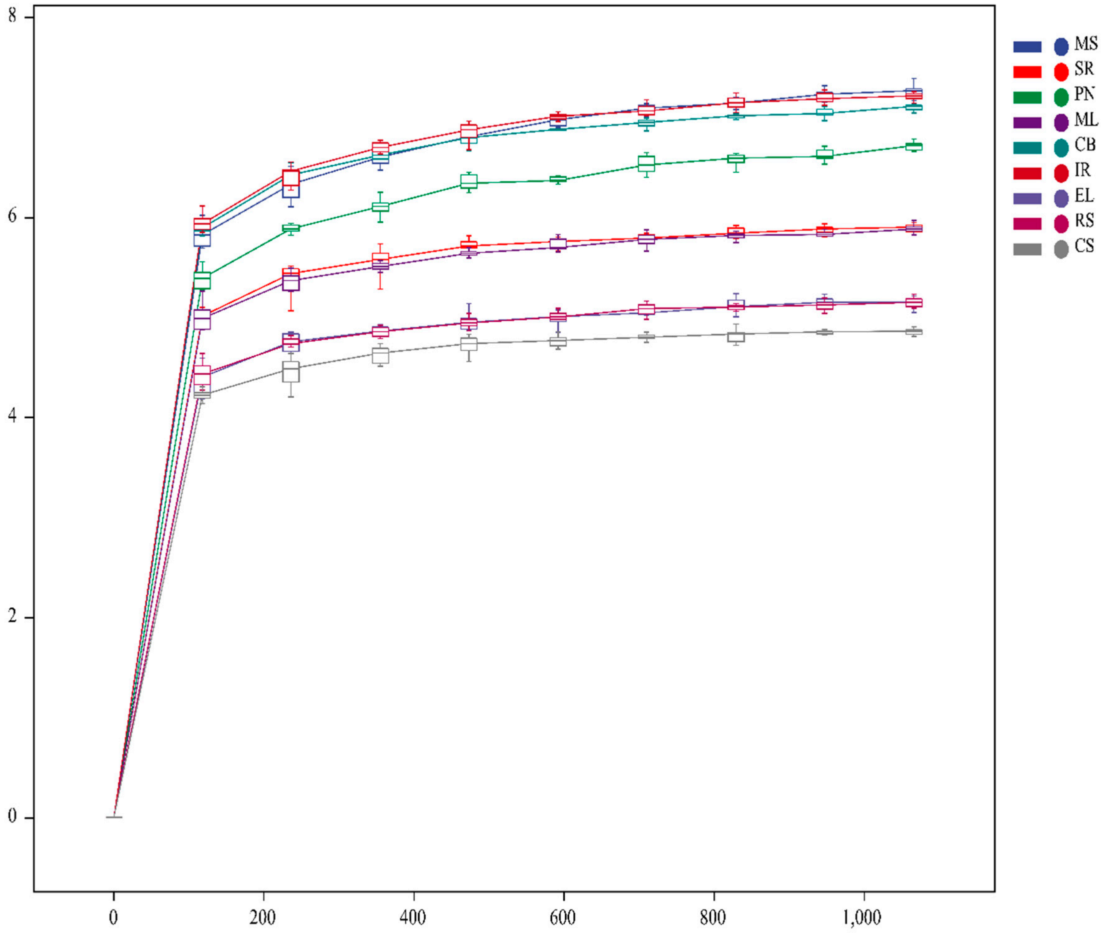

Figure S2. Assessment of sparse curves for bacterial sequencing saturation.

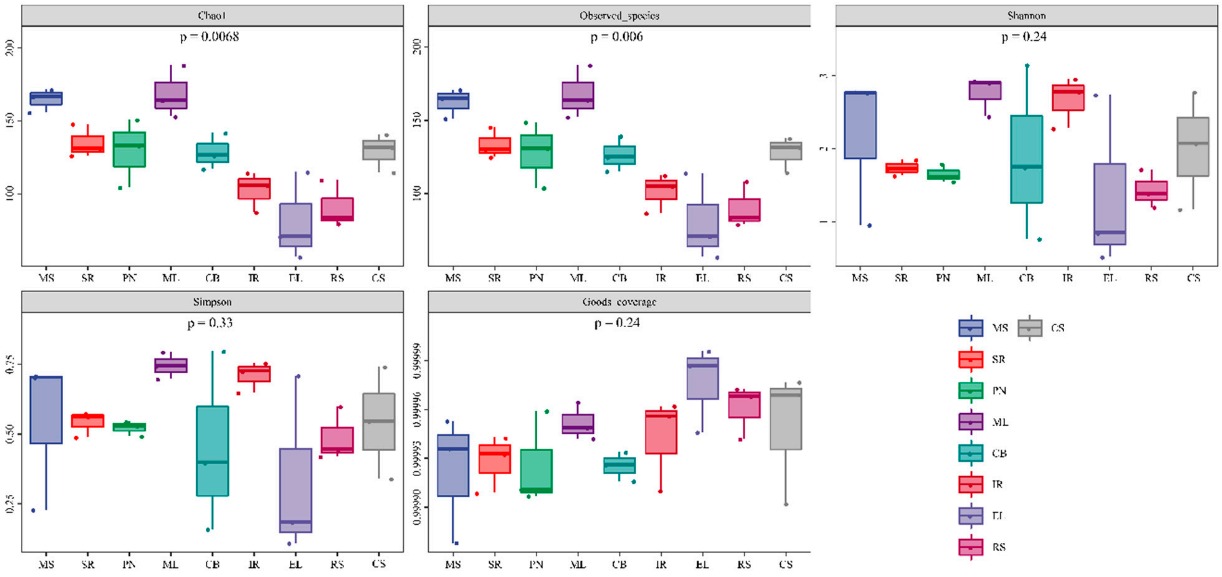

Figure S3. Alpha diversity indices for fungal communities.

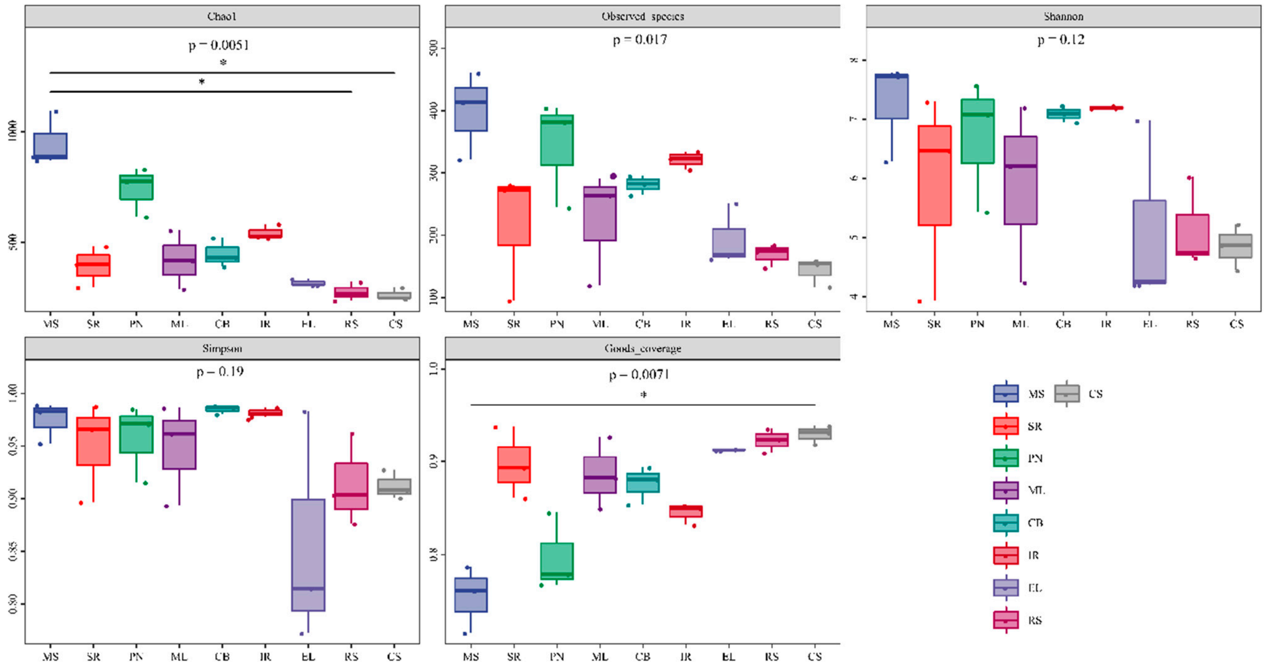

Figure S4. Alpha diversity indices for bacterial communities.

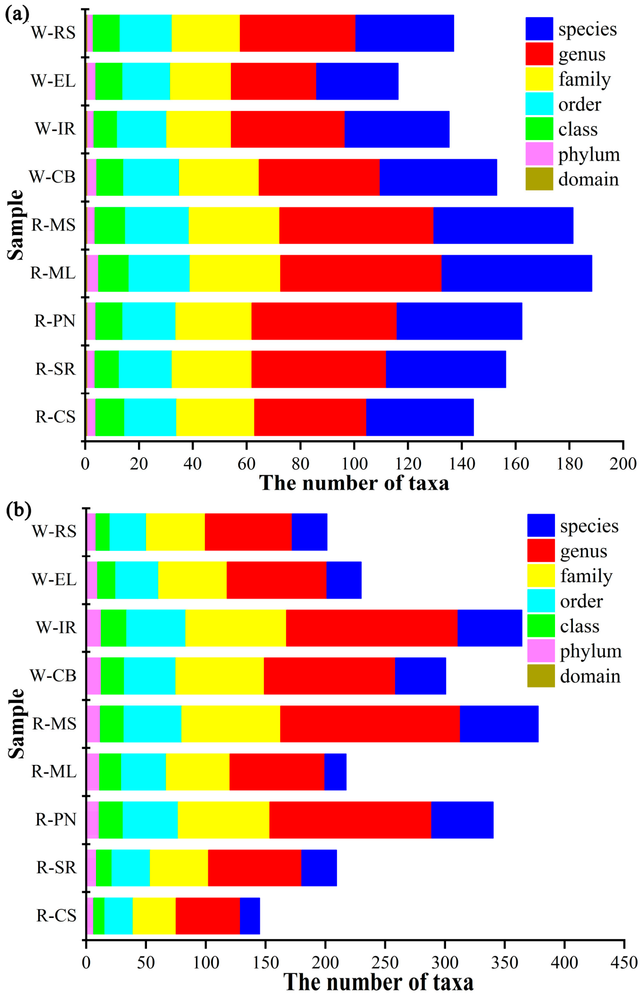

Figure S5. Statistics of the microbiota at each classification level for fungi (a) and bacteria (b).
